# Modulation of Renal Insulin Signaling Pathway and Antioxidant Enzymes with Streptozotocin-Induced Diabetes: Effects of Resveratrol

**DOI:** 10.3390/medicina55010003

**Published:** 2018-12-31

**Authors:** Gökhan Sadi, Gamze Şahin, Aykut Bostanci

**Affiliations:** Department of Biology, K.Ö. Science Faculty, Karamanoğlu Mehmetbey University, 70100 Karaman, Turkey; gokosad@gmail.com (G.Ş.); sadi@kmu.edu.tr (A.B.)

**Keywords:** diabetes kidney, oxidative stress, inflammation, resveratrol, insulin signaling

## Abstract

*Background and objectives:* Diabetes mellitus is a disease of insulin deficiency or its inability of usage by the target tissues leading to impairment of carbohydrate, lipid, and protein metabolisms. Resveratrol, having robust anti-inflammatory and anti-oxidant properties, has a high potential to treat or prevent the pathogenesis of diseases. This study was conducted to reveal the relationship between diabetes-induced oxidative stress and tissue inflammation with changes in main enzymatic antioxidants (*cat, sod, gpx,* and *gst*) and the components of the insulin signaling pathway (*insulin Rβ, irs-1, pi3k, akt, mtor*) in kidney tissues. Additionally, the effects of resveratrol on these parameters were evaluated. *Materials and Methods:* Male Wistar rats were randomly divided into four groups; (1) control/vehicle; (2) control/20 mg/kg resveratrol; (3) diabetic/vehicle; (4) diabetic/20 mg/kg resveratrol. Gene and protein expressions of antioxidant enzymes and insulin signaling elements were evaluated in renal tissues. *Results:* Downregulation of antioxidant enzymes’ gene expression in the kidney tissues of diabetic rats was demonstrated and this situation was devoted partially to the reduced gene expression of *nfκb.* Moreover, the components of renal insulin signaling elements were upregulated at both gene and protein expression levels in diabetic rats, and resveratrol treatment decreased this sensitization towards the control state. *Conclusion:* Resveratrol partially improved diabetes-induced renal oxidative stress and inflammation due to healing action on renal antioxidant enzymes and insulin signaling pathway components.

## 1. Introduction

Decreased insulin secretion and/or its responsiveness to the tissues, leading to dysfunctions of protein, lipid, and carbohydrate metabolism is characterized as diabetes mellitus [1]. Hyperglycemia is the major hallmark of the disease and associated with increased rate of glucose auto-oxidation, non-enzymatic protein glycosylation, and increase influx to the polyol pathway [2,3]. These mechanisms initiate oxidative stress and inflammation and might affect many cellular metabolic activities [4]. Prolonged oxidative stress decreases antioxidant capacity which enhances chronic complications of diabetes [5]. In fact, enzymatic and non-enzymatic defensive mechanisms reduce cellular oxidative stress in tissues. Superoxide dismutase (SOD) isozymes; SOD1 and SOD2 neutralizes superoxide radicals in cytoplasm and mitochondria, respectively. Catalase (CAT) decompose hydrogen peroxide to water a function that is shared with glutathione peroxidase (GPx). Furthermore, conjugation of glutathione to a wide range of electrophiles including oxidatively modified compounds are catalyzed with a group of enzymes called glutathione S-transferases (GSTs).

Recent studies have indicated a strong relationship between oxidative stress and steady-state levels of antioxidant enzymes [6,7]. These enzymes are also regulated by two transcription factors; nuclear factor erythroid 2-related factor (Nrf2) and nuclear factor kappa B (NFκB) [8]. Targets of these two proteins are important for the protection since they enhance proinflammatory cytokines and defense against inflammation and oxidative stress [9,10]. Additionally transcriptional, translational, and post-translational mechanisms as a consequence of changes in the cellular redox potential strongly regulate the activities or the existence of antioxidant enzymes in the cells [11]. Resveratrol (3,4,5-trihydroxystilbene) is a phytoalexin found in abundance in red colored fruits such as grapes, peanuts, strawberries, and cherries. It has strong antioxidant, anti-inflammatory, and anti-apoptotic effects [12]. Its role in protection against oxidative damage in the pathophysiology of diabetes has been demonstrated in current studies [13,14,15].

Kidney tissues play a role in glucose homeostasis through the tubular glucose reabsorption and renal gluconeogenesis [16]. They are directly involved in the pathogenesis of diabetes and understanding the renal physiology and function in diabetics has become indispensable to all specialties treating diabetic patients. In our previous studies, impaired glucose metabolism in the liver tissues leading to adverse effects on the hepatic insulin signaling pathway has been demonstrated [17]. Moreover, the regulation of main antioxidant enzymes in brain and liver tissues of diabetic rats and the effects of resveratrol on these parameters have been established [5,11]. Based on these findings, to understand molecular alterations in renal insulin signaling pathway and antioxidant systems in diabetes and in vivo effects of resveratrol, we hypothesized that diabetes-related modifications in renal tissues could be returned to normal conditions with resveratrol. To make track for the concrete molecular action mechanism of the resveratrol through the regulation of the renal function, the present study was designed to investigate the effects of diabetes and resveratrol on oxidative and inflammatory biomarkers. Additionally, regulation of renal insulin signaling pathway components and antioxidant enzymes are inspected in an animal model of streptozotocin (STZ)-induced diabetes.

## 2. Materials and Methods

### 2.1. Animal Treatments

The Committee for the Ethical Animal Care approved (Kobay DHL, 2012/45) all the animal procedures which were conducted along with the rules of the Guide for the Care and Use of Laboratory Animals (NIH publication no. 85/23, revised in 1986). Accordingly, male Wistar rats, which were eight-week-old, were housed in temperature-controlled rooms (20–22 °C) with a 12-hour light-dark cycle. They had a standard rodent diet (chow pellet) composed of 23% protein, 4% fat, 62% starch, 7% cellulose together with standard vitamins and salts. One week after acclimation, animals were randomly divided into four groups. The control group (C) (*n* = 12) were injected only vehicle; 10% dimethyl sulfoxide (DMSO) for four weeks. Resveratrol group (RSV) (*n* = 12) were administered with a daily intraperitoneal dose of resveratrol (20 mg/kg/day) in the vehicle throughout the four-week period. Diabetes group (D) (*n* = 12) received a single dose of streptozotocin (STZ) (55 mg/kg) dissolved in 0.05 M citrate buffer (pH 4.5) and daily vehicle for four weeks. Diabetes + Resveratrol group (D + RSV) (*n* = 9) were given intraperitoneal 20 mg/kg/day resveratrol throughout the four-week period, starting from two days after STZ administration. Fasting blood glucose levels were measured by Accu-check-go (Roche, Germany) glucometer weekly from the blood of tail veins. Animals having blood glucose concentration higher than 200 mg/dL were considered diabetics. All rats were decapitated at the end of the study period. After removal from the rats, kidney tissues were blotted dry and frozen quickly with liquid nitrogen. Then they were stored at −85 °C until use.

### 2.2. Tissue Homogenization and Measurement of Renal Malondialdehyde (MDA) Contents

Kidney tissues were homogenized in a medium containing Tris buffer (50 mM; pH 7,4), NaCl (150 mM), EDTA (5 mM), Triton X-100 (1% *w/w*), sodium deoxycholate (0.26% *w/v*), sodium fluoride (50 mM), sodium orthovanadate (0.1 mM) and PMSF (0.2 mM) with TissueRuptor™ homogenizer (Qiagen, Venlo, The Netherlands). After centrifugation at 1500× *g*, protein concentrations of supernatants were determined spectrophotometrically [18]. MDA levels, the end product of lipid peroxidation, were determined by HPLC with Chromsystems Diagnostics (Munich, Germany) kit and HPLC fluorescence detector (Ex: 515 Em: 553 nm).

### 2.3. Determination of Gene Expressions of Antioxidant Enzymes, Insulin Signaling Pathway Components, and nfκb with Real-Time Polymerase Chain Reaction

RNeasy total RNA isolation kit (Qiagen, Venlo, Netherlands) was utilized to isolate total RNA from the kidney tissues. Quantity and quality of isolated total RNAs were determined with spectrophotometry at 260/280 nm and agarose gel electrophoresis, respectively. cDNA synthesis was performed with 1 µg of total RNA using commercial first strand cDNA synthesis kit (Thermo Scientific, Massachusetts, USA). Gene expression levels of antioxidant enzymes and insulin signaling pathway components were determined with qRT-PCR (LightCycler 480 II, Roche, Berlin, Germany) as we described in detail previously [17,19]. Nucleotide sequences of pre-validated primer pairs are given in Table 1. Efficiency corrected advance relative quantification tool provided by the LightCycler^®^ 480 SW 1.5.1 software was utilized to determine the relative expression of genes with respect to the internal control, *gapdh* (glyceraldehyde 3-phosphate dehydrogenase).

### 2.4. Immunoblot Analysis of Antioxidant Enzymes; CAT, SOD1, SOD2 and Insulin Signaling Pathway Components; Insulin Rβ, PI3K, AKT1

CAT, SOD1, SOD2, Insulin Rβ, PI3K, AKT1 protein contents were determined by western blot analysis. Briefly, homogenates containing 50 µg of proteins were separated by SDS-PAGE and electroblotted onto PVDF membranes. Then, blotted membranes were blocked with 5% (*w/v*) non-fat dried milk and incubated with CAT (Anti-CAT Rabbit IgG, Santa Cruz Biotechnology, Santa Cruz, USA, sc-50508, 1:500), SOD1 (Anti-SOD1 Goat IgG, Callbiochem: 574597, 1:1000), SOD2 (Anti-SOD2 Rabbit IgG, Santa Cruz, sc-30080, 1/1000), Insulin Rβ (Anti- Insulin Rβ Rabbit IgG, Santa Cruz, sc-711, 1/100), PI3K (Anti-PI3K Rabbit IgG, Santa Cruz, sc-423, 1/100), AKT1 (Anti-AKT1 Rabbit IgG, Santa Cruz, sc-8312, 1/100) primary antibodies for two hours. As a cytoplasmic internal control, GAPDH proteins were also labeled with anti-GAPDH Rabbit IgG (Santa Cruz, sc-25778, 1:2000). After primary antibody incubation, horseradish peroxidase (HRP) conjugated secondary antibodies were incubated (1:10,000) for 1 h. Corresponding proteins were detected after incubation with Clarity^TM^ Western ECL (Bio-Rad Laboratories, Hercules CA, USA) substrate solution. Western blot images were gained with ChemiDoc^TM^ MP Chemiluminescence detection system (Bio-Rad Laboratories, Hercules, CA, USA). ImageLab4.1 software was operated to analyze the relative expression of proteins with respect to internal standard.

### 2.5. Determination of Antioxidant Enzyme Activities

Catalase activities were determined using a method in which the rate of H_2_O_2_ decomposition was followed at 240 nm [19]. GPx activity was determined by measuring the oxidation of NADPH at 340 nm spectrophotometrically [20]. Total GST activity in kidney tissues was determined according to the method [21] in which conjugation of 1-chloro-2,4-dinitrobenzene (CDNB), a common substrate for all GST isozymes, with reduced glutathione is followed at 340 nm. Enzyme activities were calculated as U/mg, which reflects the amount of substrate consumed in one minute by one mg protein containing the cytosolic fraction. Superoxide dismutase (SOD) activity was determined as the amount of protein that inhibits pyrogallol auto-oxidation by 50% [22].

### 2.6. Statistical Analysis

Data were expressed as mean ± standard error of means and compared for differences using the Statistical Package for Social Sciences version 21.0 (SPSS IBM, Armonk, NY, USA). Statistical comparisons were performed using one-way ANOVA followed by an appropriate posthoc test (Tukey’s honestly significant difference). *p* values <0.05 were considered as statistically significant.

## 3. Results

### 3.1. Effects of Diabetes and/or Resveratrol on Some Metabolic Parameters

Weights of diabetic animals were significantly reduced after four weeks of diabetes compared to the control group. Parallel to the increase in fasting blood sugar levels, the amount of glucose in the kidney tissues was elevated in diabetic rats (43%). Resveratrol did not significantly alter the diabetic blood glucose levels but resulted in a reduction of body weight [11]. When the metabolic effects of diabetes on kidney tissues are examined, MDA levels were significantly (*p* < 0.05) increased (2-fold) and resveratrol application to diabetic animals normalizes this situation to the control state. Additionally, significant upregulation of proinflammatory cytokines; IL-6, IL-8, and TNF-α in renal tissues of diabetic rats have been demonstrated previously [14]. In this study, we also confirmed the anti-inflammatory effects of resveratrol since inflammatory markers decreased to the control levels with resveratrol application [14].

### 3.2. Changes in Gene Expression Levels of Antioxidant Enzymes

**Figure 1:**
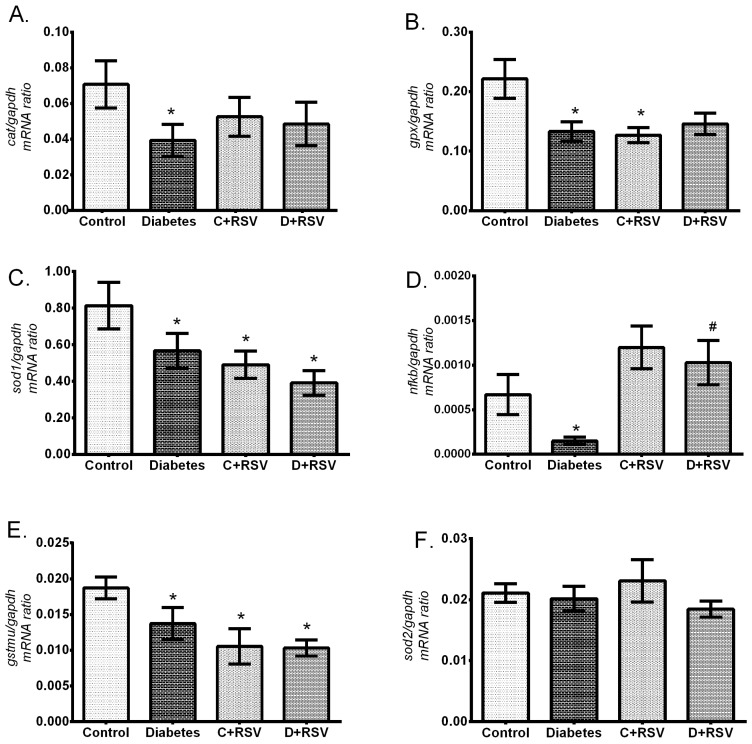
Changes in renal gene expression levels of (**A**) *cat*, (**B**) *gpx*, (**C**) *sod1*, (**D**) *sod2*, (**E**) *gstmu*, (**F**) *nfkb* with diabetes and resveratrol. Data are given with respect to *gapdh* which is used as internal standard. C: Control (*n* = 12), D: Diabetes (*n* = 12), C + RSV: Resveratrol given control (*n* = 12), D + RSV: Resveratrol given diabetic group (*n* = 9). *signifies the statistical difference of the data according to the control group (*p* < 0.05), and the # sign indicates the statistical difference of the data according to the diabetes group (*p* < 0.05).

Changes in expression levels of *gtsmu*; a biotransformation enzyme, *cat, sod1, sod2, gpx;* main antioxidant enzymes and *nfkb;* an antioxidant response element regulator were measured by qRT-PCR and the results are summarized in Figure 1. Diabetes significantly suppressed the expression levels of major antioxidant enzymes; *cat, gpx, sod-1,* and *gstmu* compared to control group (*p* < 0.05) (Figure 1A–C,E). We can explain one of the causes of this suppression, which occurs at about 40% levels, with a significant decrease (about 3-fold) in the *nfkb* (*p* = 0.033) that controls the transcription of those enzymes (Figure 1F). In addition, mRNA levels of *sod2*, which is an important mitochondrial enzyme against oxidative stress, did not change significantly with diabetes and/or resveratrol (Figure 1D). Even though, resveratrol treatment to the control group suppressed antioxidant genes; especially *gpx, sod1,* and *gstm* (*p* < 0.05), it was not effective enough to normalize the diabetic state to the control values. While resveratrol treatment to the diabetic animals was not effective on the gene expression level of antioxidant enzymes, *nfkb* got back to the control levels.

### 3.3. Changes in Protein Expression of Antioxidant Enzymes

**Figure 2:**
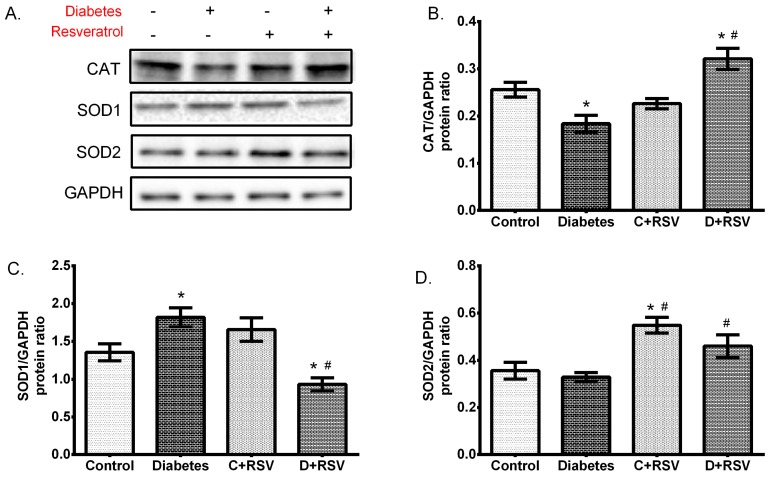
Changes in protein levels of antioxidant enzymes in diabetic and/or resveratrol-treated kidney tissues. The figure demonstrates (**A**) representative Western blot bands indicating the group averages, (**B**) changes in catalase (CAT) expression, (**C**) changes in superoxide dismutase 1 (SOD1) expression, (**D**) changes in SOD2 expression levels. Data are given with respect to glyceraldehyde 3-phosphate dehydrogenase (GAPDH) protein. C: Control (*n* = 12), D: Diabetes (*n* = 12), C + RSV: Resveratrol given control (*n* = 12), D + RSV: Resveratrol given diabetic group (*n* = 9). *signifies the statistical difference of the data according to the control group (*p* < 0.05), and the # sign indicates the statistical difference of the data according to the diabetes group (*p* < 0.05).

Western blot analysis was performed to determine whether changes in the mRNA level of the antioxidant enzymes were reflected in the amount of protein and thus, whether diabetes affects any protein translation. Figure 2 summarizes how the expression levels of CAT, SOD1, and SOD2 proteins were regulated with diabetes and/or resveratrol. Accordingly, diabetes significantly suppressed the renal CAT protein (*p* = 0.01), while the SOD1 protein levels were upregulated (*p* = 0.02) (Figure 2B,C). Resveratrol, when applied to the control group, significantly increased SOD2 protein (*p* = 0.01) but did not show the same effect to other antioxidant enzymes. As given to the diabetic animals, resveratrol normalized the changes in antioxidant enzymes. In other words, decreased CAT and increased SOD1 protein levels were normalized to the control group. The suppression of CAT protein expression correlates well with the reduction in the amount of mRNA. This shows that diabetes suppresses CAT enzyme at the transcription level in renal tissues. The change in the amount of SOD1 mRNA and protein levels with diabetes were contradictory to each other. That is, a significant decrease in mRNA levels was inversely proportional with the upregulated protein levels, indicating a post-translational activation of SOD1 with diabetes. Diabetes did not show a significant effect on SOD2 protein, whereas resveratrol application increased protein expression levels in both control and diabetic groups. This suggests a positive upregulation of SOD2 with post-translational mechanisms.

**Figure 3:**
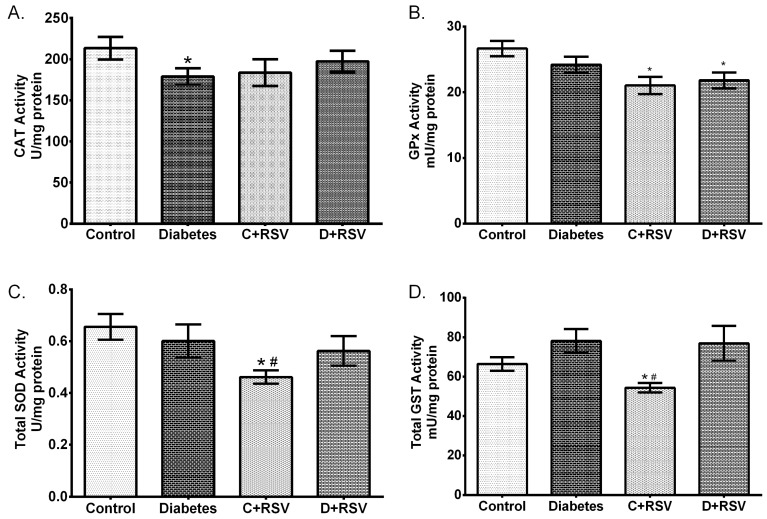
Effects of diabetes and/or resveratrol on renal (**A**) CAT enzyme activity, (**B**) glutathione peroxidase (GPx) enzyme activity, (**C**) Total SOD enzyme activity, (**D**) Total glutathione S-transferase (GST) enzyme activity. C: Control (*n* = 12), D: Diabetes (*n* = 12), C + RSV: Resveratrol given control (*n* = 12), D + RSV: Resveratrol given diabetic group (*n* = 9). *signifies the statistical difference of the data according to the control group (*p* < 0.05), and the # sign indicates the statistical difference of the data according to the diabetes group (*p* < 0.05).

After determining the changes in gene and protein expression levels, enzymatic activities which are the actual modulators of cellular oxidative stress were also determined in this study. Activity results are summarized in Figure 3. Accordingly, CAT activity was significantly suppressed with diabetes (*p* < 0.05). Suppression of CAT activity is strongly correlated with its mRNA and protein expression levels. The changes in other antioxidant enzymes with diabetes were not statistically significant. Moreover, when resveratrol was given to the control animals, GPx, total SOD, and total GST activities were downregulated significantly (*p* < 0.05).

### 3.4. Regulation of Renal Insulin Signaling Pathway Components

**Figure 4:**
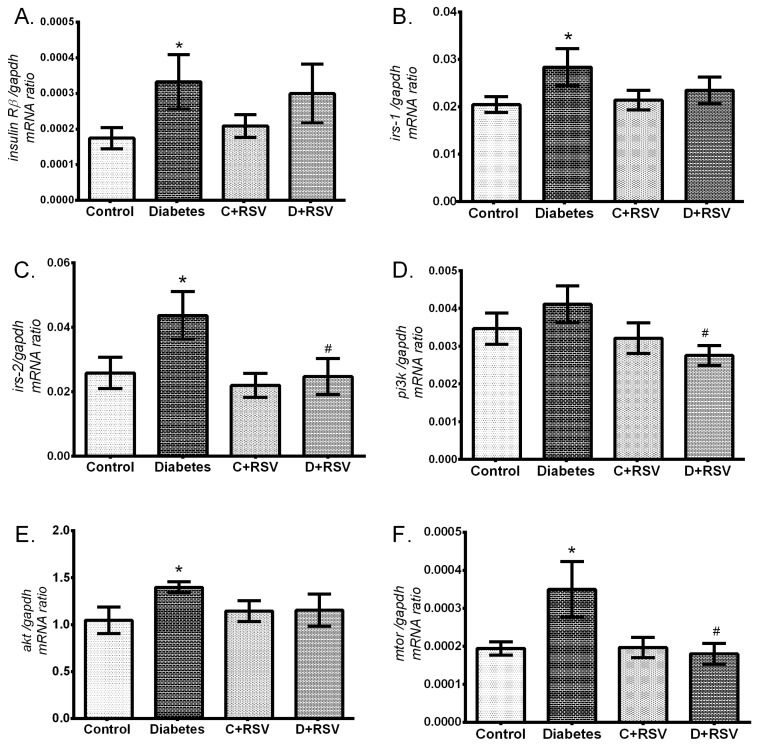
Effects of diabetes and/or resveratrol on renal (**A**) *insulin Rβ,* (**B**) *irs-1,* (**C**) *irs-2,* (**D**) *pi3k,* (**E**) *akt,* (**F**) *mtor* gene expression levels. Data are normalized with *gapdh*. C: Control (*n* = 12), D: Diabetes (*n* = 12), C + RSV: Resveratrol given control (*n* = 12), D + RSV: Resveratrol given diabetic group (*n* = 9). *signifies the statistical difference of the data according to the control group (*p* < 0.05), and the # sign indicates the statistical difference of the data according to the diabetes group (*p* < 0.05).

**Figure 5:**
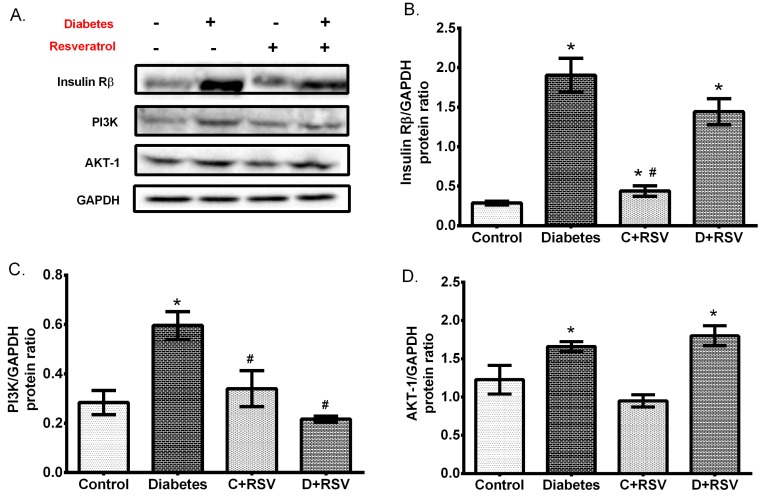
Changes in renal protein expressions of insulin signaling elements with diabetes and/or resveratrol. (**A**) Representative western blot bands indicating the group averages. Changes in (**B**) Insulin Rβ, (**C**) phosphoinositide 3-kinase (PI3K), (**D**) protein Kinase B (AKT1) protein expressions. Data were normalized with respect to corresponding GAPDH. C: Control (*n* = 12), D: Diabetes (*n* = 12), C + RSV: Resveratrol given control (*n* = 12), D + RSV: Resveratrol given diabetic group (*n* = 9). *signifies the statistical difference of the data according to the control group (*p* < 0.05), and the # sign indicates the statistical difference of the data according to the diabetes group (*p* < 0.05).

Levels of *insulin Rβ*, *irs1*, *irs2, pi3k, akt*, and *mTOR* gene expressions in renal tissues were also determined in this study. Accordingly, expression levels of genes which are involved in insulin signal transduction were all upregulated in the diabetic group (Figure 4A–E). In addition, resveratrol did not have a significant effect on renal insulin signaling when administered to control animals but reversed all the modifications towards the control values in the diabetic group. Protein expressions of insulin Rβ, PI3K, and AKT were also determined by western blotting (Figure 5). According to results, similar to gene expression levels, the insulin signaling pathway-related proteins were significantly upregulated by diabetes (Figure 5B–D). This situation suggests the activation of insulin signal transduction elements at the transcription level, resulting in a significant increase in protein expression. Furthermore, resveratrol treatment increased the insulin Rβ as applied to control animals and exerted a normalizing effect on diabetic PI3K levels in kidney tissues. Generally, it affected insulin signaling not at the protein level, but at the transcription level.

## 4. Discussion

Diabetes, usually caused by the combination of hereditary and environmental factors, results in excessive elevation of blood glucose that enhances the oxidative state in various tissues and damage to the cellular macromolecules, such as lipids, proteins, and nucleic acids [2,3]. Trans-resveratrol exhibits a wide range of biological features, including antioxidant and anti-inflammatory properties and its possible preventative and therapeutic roles in several tissues were determined previously [5,7]. Renal pathologies seem to be the most frequent complications of diabetes and new therapeutic approaches are needed in diabetic nephropathy and chronic kidney diseases. The aim of this study is to analyze the oxidative/inflammatory changes in the kidney tissues of diabetic rats and to determine the changes in antioxidant enzymes together with insulin signal transduction. Consistent with this depiction, we determined the degree of lipid peroxidation in renal tissues, which was enhanced by diabetes. We also recently published the upregulation of renal inflammatory markers; IL-6, IL-8, and TNF-α in diabetic rats [14]. All these results indicate the oxidized and inflammatory state in renal tissues of diabetic rats. Resveratrol alleviated these biomarkers toward the control levels because of its antioxidant and anti-inflammatory properties and contributed therapeutic effects on diabetic complications [23].

Superoxide dismutase is one of the most important antioxidant enzymes that convert superoxide radicals into the hydrogen peroxide. In the presence of transition metals, hydrogen peroxide might turn into hydroxyl radicals known as the most reactive species. However, CAT and GPx enzymes neutralize the hydrogen peroxide into the water in peroxisomes and cytoplasm, respectively. In addition, GSTs also play a role in cleansing toxic intermediates and eliminating harmful products caused by oxidative stress. Resveratrol has been shown not to modify the renal antioxidant systems especially SOD and CAT activities together with MDA contents [24,25]. Besides, it ameliorated the dysfunction of antioxidant enzymes, including SOD, CAT, GPx, GST, and GR catalase, in diabetic kidneys [25]. This study revealed gene expression suppression of antioxidant enzymes in STZ-induced diabetic kidney tissues and this suppression could be partially attributed to the reduction of the redox-sensitive transcription factor; *nfκb*, which particularly affects the transcription of many inflammatory and antioxidant genes [8,26]. This could be due to extensive oxidative conditions dysregulating the initiation machinery of the antioxidant enzymes transcription. Besides, destabilization of mRNA under mild oxidative state could also contribute to the suppression of antioxidant enzymes [27,28]. This apparent decrease in antioxidant enzymes is different from the results that we obtained using brain tissues [5]. In the brain, upregulation of antioxidant enzymes were devoted as an adaptation process to the moderate increase in the oxidative stress biomarkers. The effects of resveratrol on the recovery of diabetic changes in tissue antioxidant enzymes were revealed in our previous studies in which liver and brain tissues were used [5,11]. Likewise, resveratrol reduced oxidant stress and regulated gene expression of antioxidant enzymes in kidney tissues. Additionally, normalization of *nfκb* expression with resveratrol in diabetic tissues but not antioxidant enzymes designates several other effectors in the regulation of antioxidant genes. Furthermore, enhanced GST enzyme activity due to diabetes could be an adaptation to relieve the pathology of diabetes and oxidative products.

Insulin affects the intracellular metabolism by regulating several key proteins starting from its receptor on cellular membrane and line-up until several transcription factors in the nucleus. Activated insulin receptor promotes the insulin receptor substrate family proteins (IRS) which are the adaptors of insulin signal transduction. The downstream signal paths are divided into several branches and among them, PI3K/AKT/mTOR signaling is involved in many cellular processes. In this pathway, activated IRS proteins trigger phosphatidylinositol 3-kinase (PI3K) proteins and its downstream effectors such as protein kinase B (PKB or AKT) and mammalian target of rapamycin (mTOR) proteins. Activation of AKT leads to the phosphorylation of several substrates acting on gluconeogenesis and glycogenolysis. PI3K and AKT are also known to play a role in glucose transporter (GLUT4) translocation [29]. Insulin resistance, a condition often encountered in diabetes, is caused by the downregulation of all or part of the proteins involved in insulin signaling pathways, or by the inhibition (phosphorylation) of other signaling elements that inactivate the signaling pathways. Recently, we demonstrated the suppression of insulin signaling components (PI3K/AKT/mTOR) in hepatic tissues of diabetic rats [17]. In this study, we revealed significant upregulation of insulin signaling elements at both gene and protein levels in kidney tissues. This suggests that renal insulin signal transduction undergoes an activation at transcription level and leads significant increase in their protein levels. This might be due to the need for more active signaling elements in kidney tissues due to lack of insulin and/or its action. Insulin deficiency in diabetes might have stimulated kidney tissues to make them more sensitive to insulin. Resveratrol administration brings the sensitivity of insulin signaling in the kidneys to the normal values at the gene expression level; because protein levels of insulin signal transduction elements did not change significantly with resveratrol, but the level of gene expression approached to the control levels.

## 5. Conclusions

Our data point out that STZ-induced diabetes provokes oxidative damage and inflammation in renal tissues and contribute antioxidant enzymes to be regulated at gene, protein, and activity levels. Changes in antioxidant enzymes might contribute the molecular mechanisms associated with oxidative modifications in renal tissues. Additionally, diabetes-induced upregulation of the insulin signaling pathway in association with the activation of inflammatory markers led us to propose that there could be a correlation between insulin signaling and inflammation in renal tissues of diabetic rats. The findings of the present study also revealed that resveratrol may confer beneficial effects on renal functions through its influences on antioxidant enzymes and insulin signaling. Our data are consistent with the large body of literature showing beneficial health effects of resveratrol for therapeutic intervention of diabetes-induced oxidative modifications and thereby the promising results suggest potential therapeutic targets and pathways for further evaluation. This study is subject to the following limitations. First, not all the insulin signaling and other convergent transduction elements could be studied in this study to reveal complete renal molecular regulatory mechanisms in diabetes. Secondly, the lack of marked improvement in metabolic characteristics might be attributed to the relatively shorter duration of resveratrol administration. It would be better to apply resveratrol with longer and/or higher doses. Further research is warranted to clarify the beneficial effects of resveratrol on diabetes-induced renal dysfunction.

## Figures and Tables

**Table 1:** Primer pairs used in the expression analysis of antioxidant enzymes and insulin signal transduction pathway components.

Gene	Forward Primer (5′→3′)	Reverse Primer (5′→3′)
***cat***	GCGAATGGAGAGGCAGTGTAC	GAGTGACGTTGTCTTCATTAGCACTG
***gpx1***	CCACCACCGGGTCGGACATAC	CTCTCCGCGGTGGCACAGT
***sod1***	TAGCAGGACAGCAGATGAGT	GCAGAAGGCAAGCGGTGAAC
***sod2***	GCACATTAACGCGCAGATCA	AGCCTCCAGCAACTCTCCTT
***gst-mu***	AGAAGCAGAAGCCAGAGTTC	GGGGTGAGGTTGAGGAGATG
***nfκb***	GGGTCAGAGGCCAATAGAGA	CCTAGCTTTCTCTGAACTGCAAA
***insulin rβ***	GTGCTGCTCATGTCCTTAGA	AATGGTCTGTGCTCTTCGTG
***irs1***	GCCAATCTTCATCCAGTTGC	CATCGTGAAGAAGGCATAGG
***irs2***	CTACCCACTGAGCCCAAGAG	CCAGGGATGAAGCAGGACTA
***pi3k***	ATGCAACTGCCTTGCACATT	CGCCTGAAGCTGAGCAACAT
***akt1***	GAAGAAGAGCTCGCCTCCAT	GAAGGAGAAGGCCACAGGTC
***mtor***	GCAATGGGCACGAGTTTGTT	AGTGTGTTCACCAGGCCAAA
***gapdh***	TCCTTGGAGGCCATGTGGGCCAT	TGATGACATCAAGAAGGTGGTGAAG
